# Changes in the functional characteristics of tumor and normal cells after treatment with extracts of white dead-nettle

**DOI:** 10.1080/13102818.2014.989094

**Published:** 2014-12-12

**Authors:** Ralitsa Veleva, Bela Petkova, Veselina Moskova-Doumanova, Jordan Doumanov, Milena Dimitrova, Petya Koleva, Kirilka Mladenova, Svetla Petrova, Zhenya Yordanova, Veneta Kapchina-Toteva, Tanya Topouzova-Hristova

**Affiliations:** ^a^ Department of Cytology, Histology and Embryology, Faculty of Biology, Sofia University ‘St. Kl. Ohridski’, Sofia, Bulgaria; ^b^ Department of Plant Physiology, Faculty of Biology, Sofia University ‘St. Kl. Ohridski’, Sofia, Bulgaria; ^c^ Department of Biochemistry, Faculty of Biology, Sofia University ‘St. Kl. Ohridski’, Sofia, Bulgaria

**Keywords:** *Lamium album* L., plant extracts, MDCKII, A549, actin cytoskeleton, cytotoxicity

## Abstract

*Lamium album* L. is a perennial herb widely used in folk medicine. It possesses a wide spectrum of therapeutic activities (anti-inflammatory, astringent, antiseptic, antibiotic, antispasmodic, antioxidant and anti-proliferative). Preservation of medicinal plant could be done by *in vitro* propagation to avoid depletion from their natural habitat. It is important to know whether extracts from *L. album* plants grown *in vitro* possess similar properties as extracts from plants grown *in vivo*. For these reasons, it is important to examine changes in the composition of secondary metabolites during *in vitro* cultivation of the plant and how they affect the biological activity.

We used A549 human cancer cell line and normal kidney epithelial cells MDCKII (Madin–Darby canine kidney cells II) as controls in assessing the anti-cancer effect of plant extracts. To elucidate changes in some key functional characteristics, adhesion test, MTT (3-(4,5-dimethylthiazol-2-yl)-2-5-diphenyl tetrazolium bromide), transepithelial resistance (TER), immunofluorescence staining and trypan blue exclusion test were performed.

Methanol and chloroform extracts of *in vivo* and *in vitro* propagated plants affected differently cancerous and non-cancerous cells. The most pronounced differences were observed in the morphological analysis and in the cell adhesive properties. We also detected suppressed epithelial transmembrane electrical resistance of MDCK II cells, by treatment with plant extracts, compared to non-treated MDCK II cells. A549 cells did not polarize under the same conditions. Altered organization of actin filaments in both cell types were noticed suggesting that extracts from *L. album* L. change TER and actin filaments, and somehow may block cell mechanisms, leading to the polarization of MDCK II cells.

## Introduction

*Lamium album* L. (white dead-nettle) is a herb widely used in folk medicine. This is a plant rich in secondary metabolites. It contains a variety of flavonoids, iridoid glycosides, triterpenes, etc., and possesses a wide spectrum of therapeutic activities (anti-inflammatory, astringent, antiseptic, antibiotic, antispasmodic, antioxidant and anti-proliferative).[1] Laboratory studies on biological activity of several parts of the plants displayed contradictory results. Dried flowers of *Lamium album* L. possess antioxidant, anti-proliferative, mucolytic and antispasmodic activity.[2] In all *Lamium* species tested unusual high squalene content is detected, which has significant biological activity – antibacterial, antitumor and immunostimulatory effect.[1] Antiviral activity of aqueous extracts from *Lamium album* is explained by identification of the antiviral iridoid isomers of lamiridosins which acts as hepatitis C virus entry inhibitors.[3] Stimulation of human skin fibroblasts proliferation by the heptane extract of Lamii albi flos were obtained by other studies.[4] Our previous results showed significant antimicrobial activity of different extracts from *in vivo* and *in vitro* grown *L. album* L. plants.[5] Antiviral activity of the extracts was also demonstrated.[6]

The naturally growing populations of the species are currently threatened by extinction due to extensive plant picking. Preservation could be done by *in vitro* propagation of plants of interest. It is important to know whether extracts from *L. album* plants grown *in vitro* possess similar properties as extracts from plants grown *in vivo*. For these reasons, it is important to examine changes in the composition of secondary metabolites during *in vitro* cultivation of the plant and how they affect the biological activity of plant extracts. Analysis of phenolic compounds after methanol and chloroform extraction (methanol and chloroform extracts) from *in vivo* and *in vitro* propagated *Lamium album* L. was carried out using high-performance liquid chromatography (HPLC).

In this study, the effect of extracts on main functional and morphological characteristics on cancerous (A549) and noncancerous (MDCKII) cells were investigated. We showed selective negative effect of chloroform extracts from *in vitro* cultivated plants on cancer cells.

## Materials and methods

All reagents and chemicals were supplied by Sigma-Aldrich (FOT, Sofia, Bulgaria) unless otherwise stated.

### Plant material

*Lamium album* plants were grown in their natural habitat in the Lozen Mountain, Sofia, Bulgaria. The voucher specimen SO 105183 was deposited in the Herbarium of Sofia University ‘St. Kliment Ohridski’. For *in vitro* cultivation, mono-nodal 1–2 cm stem segments from the above-ground material of *in vivo* plants were thoroughly washed with tap water and sterilized by incubation in 0.1% HgCl_2_ (w/v) for 8 min followed by three washes with sterilized distilled water. For shoot and root development, the stem segments were cultivated on basal Murashige and Skoog medium (MS) medium [7] containing 3% (w/v) sucrose and 0.7 g/L agar without any supplement of growth regulators. The *in vitro* growth occurred under aseptic controlled environmental conditions (16/8 h light/dark, 60 μmol m^−2^ s^−1^ photosynthetic photon flux density, Philips TLD-33, at 25°C, and 60%–70% relative air humidity). *In vitro* propagated plants were collected and air dried after four weeks cultivation. Mature plants of *Lamium* harvested in the place mentioned above were used as *in vivo* variants. The plant materials were dried in the shade and ground in the grinder.

### Soxhlet extraction

Samples of 3 g from *in vivo* and *in vitro* powdered aerial plants of *L. album* were extracted by Soxhlet extraction with 30 mL chloroform for 8 h until full colourless and then the same plant materials were used for second extraction with methanol. Solvents were removed by rotary evaporation and drying. Extracts were concentrated, dried and kept in darkness at 4 °C for further experiments. Completely dried extracts were dissolved in 1 mL methanol (for analysis by HPLC) or in 200–500 μL dimethylsulfoxide (DMSO) (as stock for preparing cell-treatment medium). Final concentration of extracts in medium was estimated as mg of dried extracts in mL of culture medium, final concentration of DMSO in working solutions did not exceed 2%.

### Analysis of phenolic compounds by HPLC

Extracts were dissolved in 1 mL of methanol and the analysis of phenolic compounds was carried out using a HPLC system consisting of Waters 1525 Binary Pump and Waters 2487 Dual UV-vis Absorbance Detector. The separation was performed on Discovery HS C18 (25 cm × 4.6 mm × 5 μm) column with mobile phases as follows: (1) 2% acetic acid and 0.5% acetic acid/acetonitrile 95:5 (v/v) for phenolic acids; (2) 2% acetic acid and methanol 70:30 (v/v) for flavonoids; (3) 2% acetic acid and acetonitrile 80:20 (v/v) for quercetin glycosides and (4) methanol, sulphuric acid and water 50:0.3:49.7 (v/v) for rosmarinic acid. The samples (20 μL) were injected and the flow rate of the elution was 1 mL/min. Chromatograms were monitored as follows: 280 and 320 nm for phenolic acids, 380 and 308 nm for flavonoids, 370 nm for quercetin glycosides and 327 nm for rosmarinic acid. Identification of phenolic acids was based on retention times in comparison with standards. The quantification was carried out using the external standards method. Stock solution of standard compounds at concentration 1 mg/mL each was prepared in methanol, and several dilutions with mobile phase were made. The solution of standards at various concentrations (0.005, 0.010, 0.020, 0.050 and 0.100 mg/mL) was injected into the HPLC system and the calibration curves were established for each standard compound. The concentration of the compound was calculated from peak area according to the calibration curves. The amount of each phenolic acid was expressed as milligram per gram of dry weight (mg g^−1^ DW).

### Cell cultures

We used a human cancer cell line – lung carcinoma A549, and normal kidney epithelial cells – Madin–Darby canine kidney cells II (MDCKII) as controls in assessing the anti-cancer effect of plant extracts. Human lung cell line A549 was purchased from Bulgarian National Bank for Industrial Microorganisms and Cell Cultures. Cells were grown in 25 cm^2^ ‘CELLSTAR®’ flasks, at standard conditions in humidified atmosphere with 5% СО_2_, at 310.15 °K (37 °C), in Dulbecco's modified eagle medium (DMEM), supplemented with 10% fetal bovine serum (FBS) (BioWhittaker™) and 1% (v/v) antibiotic–antimycotic solution (penicillin 100 U/mL, streptomycin 100 μg/mL and amphotericin B 0.25 μg/mL, BioWhittaker™). Cells were plated on 24-well plates ‘Nunk™’, sterile cover slips, on transwell filters or 35 mm ‘CELLSTAR®’ Petri dishes, depending on the tests to be performed. Treatment was carried out for 24 or 48 h with plant extracts diluted in the culture medium at final concentration of 1 mg/mL.

### Morphological and functional tests

#### Cell viability test

The viability of cells was assessed by MTT (3-(4,5-dimethylthiazol-2-yl)-2-5-diphenyl tetrazolium bromide) assay. This approach is based on the reduction of MTT by the mitochondrial dehydrogenase of intact cells to a purple formazan product. Cells (3 × 10^4^ cell/mL) were routinely plated in 35 mm ‘CELLSTAR®’ Petri dishes at 37 °C for 24 h prior to use. After 24 h, the medium was replaced with new, containing different amounts and combinations of extracts. On the 24th/48th hour, this medium was carefully removed by aspiration. 800 μL of 0.5mg/mL MTT reagent in culture media was added to each Petri dish and incubated for 3 h at 37 °C. After removal of the MTT reagent, an equal amount (800 μL) of 10% SDS in 0.01 mol/L HCl solution was added in order to dissolve formed formazan crystals overnight at 37°C. The intensity of yellow colouration was determined spectrophotometrically λ = 570 nm, by using Boeco S-22 UV/VIS Spectrometer and calculated as per cent of control (cells incubated at the same conditions, but without extracts). EC50 was determined as a concentration of extracts, showing 50% viability of the cells, comparing to the control.

#### Test for cell adhesion

Cells were re-suspended with 1 × 10^4^ cell/mL in cultural media, with different concentration of extracts and seeded in 24-well cultural plates ‘NunkTM’. In every 60 min, cells from one well per sample were collected and number of unattached cells was determined by counting on haemocytometer. Results were determined as a per cent of cells seeded in the well.

#### Assessment of membrane permeability with a modified trypan blue exclusion assay

Loss of the integrity of plasma membrane can be demonstrated using polar dyes, such as trypan blue, which are excluded by an intact membrane. After incubation, the samples were washed three times with phosphate buffer saline (PBS) to remove the incubation medium, and stained *in vivo* with 200 μL 0.8% trypan blue in PBS per well. Negative control was alveolar cells cultivated in the same plate without plant extracts. As a positive control for membrane permeabilization, A549 cells permeabilized 5 min with 0.5% Tween 20 were used. Each sample was observed under Olympus inverted microscope and several microphotographs of each well were taken immediately after staining.

#### Methylene blue staining for evaluation of cell morphology

The cells, cultured on sterile cover slips, were treated with plant extracts for 24 h. The samples were fixed with phosphate-buffered formalin for 20 min and stained with 1% Methylene blue in PBS for 5 min, dehydrated and mounted with Canada balsam. Cell morphology was observed under light microscope Olympus CX21.

#### Transepithelial resistance (TER)

To determine changes in the polarization of MDCK II cells in result of treatment with different *Lamium album* L. extracts, we investigated the TER by specially designed voltmeter EVOM. MDCK II cells were grown on six-well transwell Corning filters (initial concentration of 5 × 10^4^ cells per transwell filter) for 10 days in medium, containing 1 mg/mL different extracts of *Lamium album* L. The TER was measured for 8 and 10 days at 24-h intervals by voltmeter EVOM. Cell polarity was measured when extracts were administered at the beginning of cultivation and when administration was at the fourth day of cultivation, corresponding to the beginning of polarization. Measurement of the ion conductivity through the membrane is a good indicator for the level of cell polarization.

#### Fluorescence staining of actin

A549 and MDCK II cells were grown on cover slips for two days with initial concentration of 1 × 10^5^ cells/well. The cells were washed with 1× PBS (containing 100μmol/L CaCl_2_ and 1mmol/L MgCl_2_), fixed for 15 min with 4% formaldehyde and permeabilized with 5% Tween 20 in PBS for 10 min. Cells were stained for 45 min with phalloidin, conjugated with TRITC (Sigma-Aldrich) and were visualized with Nikon TiU confocal laser scanning microscope and the images were acquired and processed using EZC1 software.

#### Statistics

All obtained data were statistically evaluated using GraphPad InStat 3 Software. *P* < 0.05 (*) *P* < 0.01 (**) and *P* < 0.001 (***) were accepted as statistically significant.

## Results and discussion

**Figure 1.**
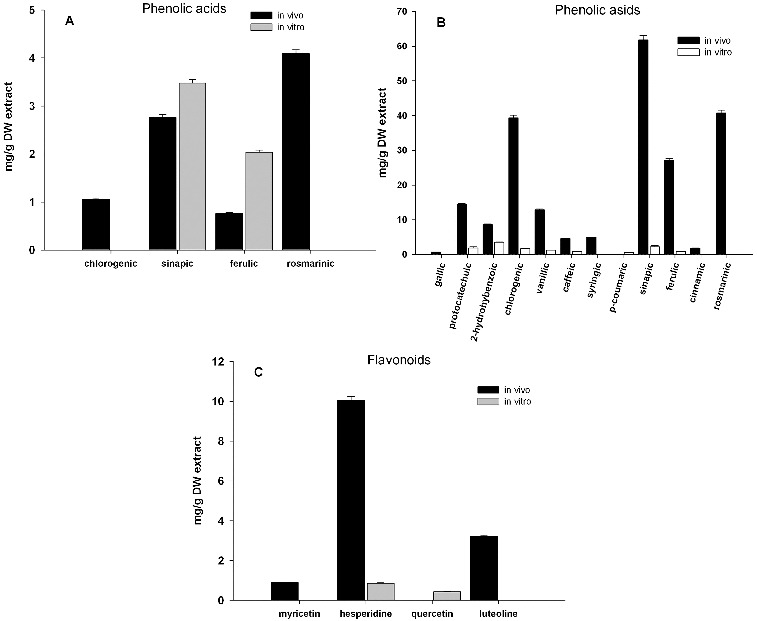
Content of phenolic acids from chloroform (A) and methanol (B) extracts and flavonoids (C) from methanol extracts of *in vivo* and *in vitro* propagated plants of *Lamium album* L. LaMeS *in vivo* – methanol extracts from *in vivo L. album*; LaChlS *in vivo* – chloroform extracts from *in vivo L. album*; LaMeS *in vitro* – methanol extracts from *in vitro L. album*; LaChlS *in vitro* – chloroform extracts from *in vitro* propagated *L. album*.

Analysis of hydrophobic compounds in the plant extract detected by HPLC-UV showed differences between *in vitro* cultivated plants and those collected from natural deposits (referred as *in vivo* plants). Twelve phenolic acids in methanol extracts and considerably less in chloroform extracts were identified from both *in vivo* and *in vitro* plants of *L. album* (Figure 1(A) and 1(B)). *In vitro* cultivated plants had significantly less compounds. The most distinct differences were observed in content of chlorogenic, sinapic ferulic and rosmarinic acids (Figure 1).

Methanol extracts were characterized with respect to both phenolic acids and flavonoid content. The result showed that the extract of *in vivo* plants was a very rich source of phenolic acids, most abundant being chlorogenic (39.3 mg g^−1^), sinapic (61.8 mg g^−1^) and rosmarinic acid (40.8 mg g^−1^), followed by ferulic, vanillic and protocatechuic acids (Figure 1(B)). These antioxidants, especially the rosmarinic acid, possess several important biological properties, including antioxidant, antibacterial, antiviral and anti-inflammatory activities.[8] Antihyperglycemic properties have been recently reported for sinapic acid.[9] Among the four tested flavonoids detected in the methanolic extracts, three were accumulated mainly in the *in vivo* plants (Figure 1(C)). The main one was hesperidine, a flavonoid typical for citrus fruits, which was found to exert a wide range of pharmacological effects.[10]

*In vitro* plants accumulated considerably less phenolic acids and flavonoids. It is interesting to note that the main phenolic acid in *in vitro* cultures was the well-known 2-hydroxybenzoic (salicylic) acid. Only two flavonoids were detected in methanol extract and only two phenolic acids (sinapic and ferulic) in the chloroform extracts (Figure 1).

In order to assess potential anti-cancer effect of *Lamium album* L. extracts, we performed several *in vitro* tests on two mammalian cell cultures – a carcinoma cell line A549 and a non-cancerous cell line MDCKII. The influence of the extraction solvents on the cells is negligible, because the resulting extracts were dried completely before they can be dissolved in an intermediate solvent (DMSO), and then in a culture medium. First, we checked whether changes in cell morphology occurred during the incubation of the cells with media containing plant extracts.

**Figure 2.**
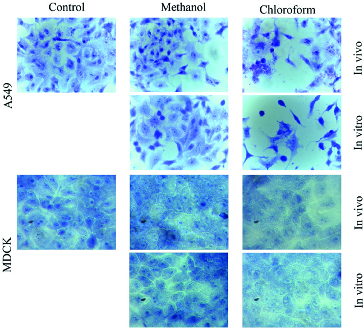
Morphological changes in A549 and MDCKII cells after 24-h treatment with methanol (Me) and chloroform (Chl) extracts from *in vivo* and *in vitro* propagated *Lamium album* L. plants.

Methanol and chloroform extracts of *in vivo* and *in vitro* propagated plants affected differently cancerous and non-cancerous cells. The most significant changes were observed in cancer cells incubated with chloroform extracts (Figure 2). A549 cells were shrunk, with disrupted intercellular contacts and damaged cytoplasm. In some cases, nuclear anomalies (bi-nucleated cells, fragmentation of nuclei, etc.) were observed. The MDCKII cells were unchanged or slightly affected compared to the cancerous cell line.

The cytotoxic effect on cancer cells can be explained by the higher content of sinapic acid in chloroform extracts (Figure 1(A)). It has been shown in other plant extracts that this acid has histone deacetylase inhibitory activity and anti-proliferative activity on HeLa cells. [11] The slight influence of methanolic extracts could be explained by a compensatory effect of the remaining phenolic acids or their combination. Similar effects (cardioprotective and antioxidant) of Chinese medicinal plant extracts were reported by Li et al.[12]

In our previous studies, we investigated potential effects of several concentrations and combinations of methanol and chloroform extracts from *in vivo* and *in vitro* propagated *Lamium album* L. on A549 cancer lung cell line, and observed reduction in cell viability and reduction of adhesion properties of the cells.[13] Cell adhesion is a crucial process in the metastasis of tumour cells.[14] On the other hand, many normal cells must adhere to their neighbours or extracellular matrix to be able to realize their functions.

Significant suppression of the adhesion of A549 cells was detected in concentration 1 mg/mL of chloroform extracts (Table 1). Up to 3 h of treatment with methanol extracts from both *in vitro* and *in vivo* propagated plants, approximately 50% of the cells were unattached. After 6 h, for *in vivo* and *in vitro* extracts, 20% and 30% of cells remain without adhesion, respectively. Extracts from *in vitro* propagated plants showed stronger effect on the inhibition of cell adhesion compared to these *in vivo*. Similar results were obtained by the chloroform extracts. The only difference was in the dynamics of the cell adhesion – methanol extracts suppress the velocity of this process, while the chloroform extracts did not change the velocity (data not shown). The results of the adhesion test with MDCK II cells showed a weak effect of the extracts compared to the A549, which was probably due to reversible changes of the adhesive complexes (Table 1).

**Table 1.** Effect of treatment with chloroform extracts on adhesion properties of cells.

Extract	A549 cells	MDCK II cells
Chloroform *in vivo*	About 25% suppressed	Unchanged
Chloroform *in vitro*	About 30% suppressed	Unchanged

**Figure 3.**
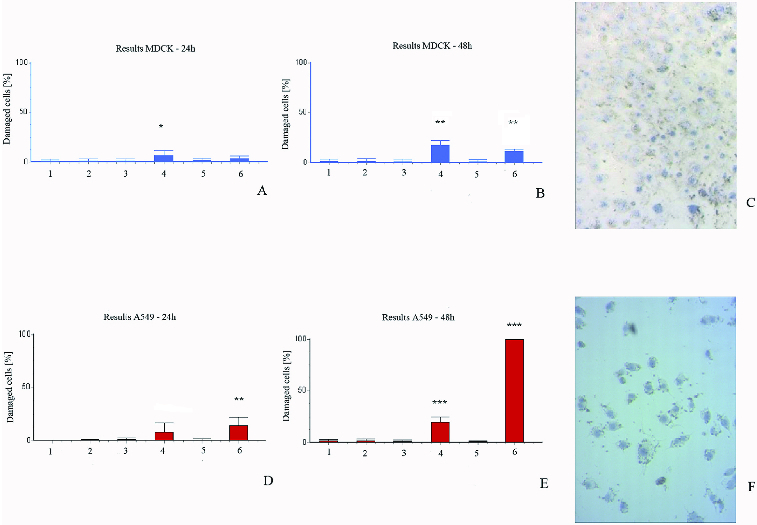
Membrane permeability test with trypan blue exclusion. MDCKII cells (A, B and C) and A549 cells (D, E and F) after 24 h (A and D) and 48 h (B, C, E and F) treatment. 1 – control; 2 – control with DMSO; 3 – cells with methanol extracts from *in vivo* plants; 4 – cells with chloroform extracts from *in vivo* plants; 5 – cells with methanol extracts from *in vitro* plants; 6 – cells with chloroform extracts from *in vitro* plants.

Surprising results were received in the membrane permeability test. In both types of cells, methanol extract did not affect membrane permeability, while chloroform extracts affected it greatly (Figure 3). In cancer cells, the most prominent permeabilization was observed upon treatment with extracts from *in vitro* cultured plants, while the normal cells were most heavily impacted by the chloroform extracts from *in vivo* plants. The initial membrane damage of cancer cells was significantly intensified at 48 h, which is a strong indication of the potential anti-cancer effect of the chloroform extracts from *in vitro* propagated plants.

In order to obtain additional information about the influence of different extracts from *Lamium album* L. on some other cell characteristics, we investigated changes in the polarity of MDCKII cells, after incubation in medium containing plant extracts. Cells from this line can reach their polarization just for five–six days growing on transwell filters, a relatively short period, making them good model for investigation of protein sorting and cell polarization.[15]

**Figure 4.**
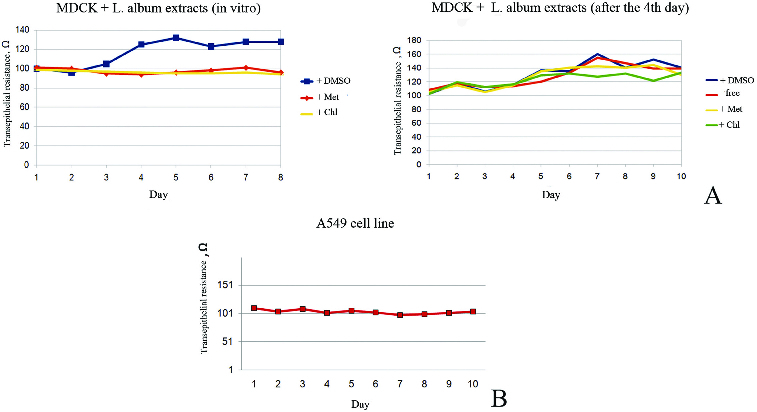
Determination of TER of MDCK II (A) and A549 (B) cells. The figure shows arrest of TER of cells after administration of different *Lamium album* L. extracts in concentration 1 mg/ml. Met – methanol extracts; Chl – chloroform extracts; DMSO – control with DMSO; free – control without DMSO.

We observed altered epithelial transmembrane electrical resistance of MDCK II cells, treated with plant extracts, compared with non-treated MDCK II cells. The first few days, non-treated cells showed low levels of resistance, corresponding to the non-polarized state. From the fifth day the resistance raise, corroborating to the polarization of the cells (Figure 4). Administration of the plant extracts at the beginning of cultivation erases this increasing of resistance, indicating alteration in the polarization mechanisms of the cells (Figure 4(A)). Similar effect of alteration was observed when extracts were administered on the fourth day of cultivation. Cancerous A549 cells did not polarized under same conditions (Figure 4(B)).

**Figure 5.**
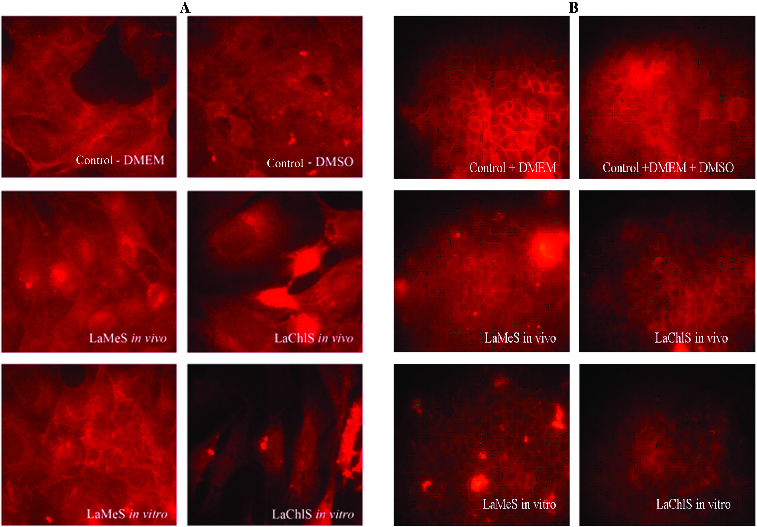
Changes in actin cytoskeleton of A549 (A) and MDCK II (B) cells. Fluorescence staining with phalloidin–TRITC, fluorescent microscopy, original magnification 600× (A) and 400× (B). control-DMEM – control without DMSO; control-DMEM+DMSO – control cells with DMSO; LaMeS *in vivo* – methanol extracts from *in vivo L. album*; LaChlS *in vivo* – chloroform extracts from *in vivo L. album*; LaMeS *in vitro* – methanol extracts from *in vitro L. album*; LaChlS *in vitro* – chloroform extracts from *in vitro L. album*.

To reinforce our results, we also investigate changes in the actin cytoskeleton. Proper organization of actin cytoskeleton is responsible for the correct protein sorting and polarization. Fluorescence staining (Figure 5) revealed altered organization of actin filaments in cells treated with plant extracts.

Notable disruption of the actin network of A549 cancer cells was detected after administration of chloroform extracts from *Lamium album* L. (Figure 5(A)). Methanol extracts did not affect these cells.

Actin cytoskeleton of non-cancer cells was slightly affected by *in vivo* extracts. The extracts from *in vitro* propagated cells had stronger effect on MDCK II cells. The actin network appeared loose, confirming the results of TER measuring (Figure 5(B)).

## Conclusions

*In vitro* cultivated *Lamium album* plants had significantly less compounds compared with plants grown in their natural habitat. Methanol and chloroform extracts of *in vivo* and *in vitro* propagated plants affected differently cancerous and non-cancerous cells. Chloroform extracts from *in vitro* propagated plants possessed a potential anti-cancer effect. These extracts affected strongly cellular membrane permeability, cell adhesion properties and morphology of cancer cells. In addition, our results suggested that both extracts change cell polarity and actin filaments, and somehow block cell mechanisms, leading to the polarization of MDCK II cells.
